# Electricity in Gynecology at the Howard Hospital, Philadelphia*Read before the Philadelphia County Medical Society, September 9, 1896.

**Published:** 1896-10

**Authors:** G. Betton Massey

**Affiliations:** Philadelphia


### ELECTRICITY IN GYNECOLOGY AT THE HOWARD HOSPITAL, PHILADELPHIA.*

G. BETTON MASSEY, M.D.,

PHILADELPHIA.

At a time when the attention of practitioners specially interested in the diseases of women is so largely occupied with the mere operative treatment of these affections, it is worthy of note, that through the liberality of the managers of the Howard Hospital in this city, a complete equipment has been provided for the application of electricity to suitable cases of these affections, affording an opportunity for the worthy poor to obtain the benefit of this remedial agency in the dispensary and wards, as well as pay patients in the private rooms. The result has been a distinct broadening of the scientific and philanthropic work of an institution that is well represented by able practitioners in the other departments of medical work.

By way of preface to this brief review of it, I may say that the electro-therapeutic work at this institution, has not been conducted with any hostility to other modes of treatment, commonly employed in similar cases, though a special effort has been made to show its comparative value in certain affections in the interests of both science and humanity; and particularly, it should be said that no pretension has been made that it is adapted to the cure of all the diseases of women. It is but a helpmate to the other remedial agencies of a well-equipped general hospital, the youngest helpmate, in fact, yet dealing, as it does, with a force that is the latest and most valuable acquisition of mankind, has demanded the unremitting attention of special workers, that knowledge and skill already acquired may be usefully applied and new scientific facts discovered. That its practice trenches upon the alleged field of other workers is a mere assumption on their part. There are no preserves in the practice of medicine that can successfully resist the inroads of a better form of treatment, and no truly scientific workers will set up any other standard of action than the best interests of the patients confined to their care.

The cases accepted as appropriately treated by electricity in this department have been strictly gynecologic, the majority presenting evidences of chronic inflammations, exudations or hypertrophies of the pelvic organs, neoplasms, pathologic displacements or disturbed

*Read before the Philadelphia County Medical Society, September 9, 1896.

innervation, including certain neuroses with pelvic manifestations and post-operative, painful affections.

During the eight years that this work has been conducted a considerable experience has been accumulated, leading to conclusions that may be of general value when summed up in concise form.

*Fibroid Tumors.*—Thirty cases of fibroid tumors of the uterus have been under treatment for varying periods, including several now in attendance. The results accomplished have been reasonably good, considering the roving nature and imperfect intelligence of the majority of clinic patients, many of whom fail to attend regularly, or stop altogether after their discomfort has been removed. A submucous tumor in one case was expelled from the uterus by electrically-induced contractions. Three tumors disappeared almost entirely by absorption, one of them so large as to fill the abdominal cavity below the navel. In eight cases there were symptomatic cure and great reduction in size. In another eight cases symptomatic cure and slight reduction in size were the result. In two cases there was cure of the symptoms, but no reduction in size. Four cases are still under treatment with a favorable outlook. In one case only was there no improvement. Three cases failed to continue the treatment after so slight a trial of the method as to leave the possibility of benefit undetermined.

Including the symptomatic cures of tumors that were arrested in growth, with or without reduction in size, the list shows that twenty-two cases were practically successful. As seven are still under treatment or were lost sight of after insufficient treatment, we have twenty-two practical successes, contrasted with but one known failure to improve, or about ninety-five and one-half per cent. of successes and four and one-half per cent. of failures.

No bad results whatever attended the treatment of any one of the cases, and practically no discomfort attended or followed any of the applications. Two cases were, however, operated upon by colleagues after the cessation of treatment, one of these, tabulated as the only case receiving no improvement after a proper length of treatment, dying under the knife, and the other, tabulated as receiving insufficient treatment, being successfully operated upon by Dr Hamill.

An impartial review of these cases cannot do otherwise than convince the unprejudiced that the electric control of the nutrition and forced absorption of fibroid tumors may be brought about in a considerable proportion of cases, and that a surgeon is not warranted in subjecting a case of this nature to the dangers and uncertain consequences of an operation for removal until the possibilities of the Apostoli treatment have been fully tested. The conditions which contraindicate the Apostoli method have now been fully defined, and include purulent formations in the tumor or its neighborhood, and cystic degeneration of the tumor itself. The existence of cystic degeneration is easily ascertained by palpation, and purulent inflammations of the adnexa are now also capable of being predicted with great certainty by means of a special diagnostic response to electric treatment, which has been pointed out by Apostoli. It has been ascertained that if the faradic current fails to relieve temporarily the pain complained of, and if the pain is aggravated by an intra-uterine galvanic application, the existence of a sub-acute purulent inflammation may be predicted with confidence. In such cases electricity intelligently applied becomes of great service to surgery in demonstrating the necessity for an early and thorough operation for the removal of a tumor that is unadapted to any other treatment. The one case mentioned as not relieved in any way by electric treatment gave distinct responses of this nature, intense cramp-like pain being provoked by intra-uterine treatment. At the operation an ovarian abscess was found on one side and a cystic ovary on the other, both presumably of gonorrheal origin.

The treatment of some of these cases was tedious, and in the end some of the twenty-two successes have bunches of harmless, fibrous tissue still remaining in the uterus, but each one of these patients presents a final result infinitely superior to the results attained in successful cases of hysterectomy for fibroid tumors in the following particulars, aside from the fact that their lives have not been placed in jeopardy; they still possess their ovaries and the womanly functions dependent on these organs; they have no abdominal scar, with existing or threatened hernia at its site to render their condition about as miserable as before seeking relief, and they have had no stump left in the abdomen to become attached to the intestines to produce after-pains and constipation.

*Chronic Metritis.*—Of a large number of cases of chronic metritis, or hyperplasia consequent upon catarrhal inflammation of the uterine cavity, sixteen cases are recorded in the hospital books as receiving electric treatment. Of these as many as thirteen were cured, two improved, and one not improved. The cured cases reported complete relief from pain and bearing-down sensations, the uterus was found to be shrunken to near or quite its normal size, and some of them reported subsequent pregnancies.

*Chronic Perimetric Inflammation.*—Fourteen cases of chronic perimetric inflammation, that is, cases in which the uterus was enlarged, fixed, and surrounded by inflamed and adherent tubes and ovaries, were under treatment, six of which were cured, six improved, and two not improved.

*Chronic Ovaritis.*—Of the same general nature were four cases of ovaritis, two of which were cured and two improved.

These three groups of cases constitute a very important portion of the affections for which parous women seek medical help, and when it is remembered that a common pathologic factor enters into them all—simply an arrested resolution following a microbic invasion, or germ-phagocytic contest, it is clearly evident that a line of treatment is indicated for all that will arouse dorman nutritive energies and assist the local tissue-scavengers, the phagoctyes and lymphatics, in reconquering the territories in which they have been so far defeated. Embryonal cell-proliferation and tissue-degradation are but other names for the debris and disorganization of this drawn battle, and it behooves us to step in and assist in reorganization rather than lop off these territories in hasty despair.

*Post-Operative Pain.*—The next largest number of cases under treatment were suffering, strangely enough, from that new disease, with which many American women are now destined to spend the remainder of their days—post-operative pain and neuroses. It is a sad warning that eight cases applied for treatment for pains, aches, and ill health following removal of the ovaries or removal of tumors, in each case the condition being worse than before the operation. From two to five years had elapsed since the operation in each case, eliminating the mere climacteric symptoms that all have to go through. One of these cases was entirely relieved, three improved, and four remained unimproved.

*Carcinoma.*—One case of incipient, but undoubted, carcinoma of the breast was cured and has remained well. Two cases of carcinoma of the cervix were temporarily improved and one not improved. The value of zinc-amalgam cataphoresis is only now being tested in this affection, and it is my intention to make a full trial of the method in carcinomata that are strictly local.

The remaining cases of interest may be reported in tabular form:

| | Cases. | Cured. | Improved. | Became Worse. |
|---|---|---|---|---|
| Subinvolution | 7 | 7 | | |
| Menorrhagia | 5 | 4 | 1 | |
| Endometritis | 4 | 3 | 1 | |
| Retroflexion | 2 | | 2 | |
| Retroversion | 2 | 1 | 1 | |
| Pyosalpinx | 2 | 1 | | 1 |
| Hydrosalpinx | 1 | 1 | | |
| Ectopic Gestation | 1 | 1 | | |
| Prolapse | 1 | | 1 | |
| Urethral Caruncle | 1 | 1 | | |
| Pruritus | 1 | 1 | | |

